# Molecular Mechanisms of Resistance and Treatment Efficacy of Delamanid Against *Mycobacterium tuberculosis*: A Systematic Review

**DOI:** 10.1002/hsr2.72481

**Published:** 2026-05-04

**Authors:** Md. Mahmudul Islam, Md. Zahid Hasan, Md. Touki Tahamid Tusar, Md. Yeamin Hossain, Md. Motaher Hossain, Md. Abdulla Al Jubayed, Md. Jubaer‐Al‐Abedin, Sheikh Soikot, Shanzida Akther, Jahid Bhuyian, Hafizur Rahman Gazi, B. M. Mahmudul Hasan, Md. Shofiul Azam, Md. Enamul Haque, Abdullah‐Al‐Jubayer, Md. Faruk Hasan, F. M. Ali Haydar, Md. Khalekuzzaman, Md. Torequl Islam, Sohel Hasan

**Affiliations:** ^1^ Department of Genetic Engineering and Biotechnology Daffodil International University Dhaka Bangladesh; ^2^ Department of Microbiology, Shaheed Shamsuzzoha Institute of Biosciences Affiliated With University of Rajshahi Rajshahi Bangladesh; ^3^ Department of Biochemistry and Molecular Biology Gopalganj Science and Technology University Gopalganj Bangladesh; ^4^ Department of Microbiology, Rajshahi Institute of Biosciences Affiliated With University of Rajshahi Rajshahi Bangladesh; ^5^ Department of Microbiology and Hygiene Bangladesh Agricultural University Mymensingh Bangladesh; ^6^ Department of Fisheries University of Rajshahi Rajshahi Bangladesh; ^7^ Department of Pharmacy University of Rajshahi Rajshahi Bangladesh; ^8^ Department of Agriculture, Rajshahi Institute of Biosciences Affiliated With University of Rajshahi Rajshahi Bangladesh; ^9^ Department of Biotechnology and Genetic Engineering Gopalganj Science and Technology University Gopalganj Bangladesh; ^10^ Department of Food and Nutrition, Barishal Home Economics College Affiliated by University of Dhaka Dhaka Bangladesh; ^11^ Department of Food Engineering Dhaka University of Engineering & Technology Gazipur Bangladesh; ^12^ Department of Microbiology University of Rajshahi Bangladesh; ^13^ Department of Botany University of Rajshahi Rajshahi Bangladesh; ^14^ Department of Genetic Engineering and Biotechnology University of Rajshahi Rajshahi Bangladesh; ^15^ Department of Pharmacy Gopalganj Science and Technology University Gopalganj Bangladesh; ^16^ Bioinformatics and Drug Innovation Laboratory BioLuster Research Center Ltd. Gopalganj Bangladesh; ^17^ Department of Biochemistry and Molecular Biology University of Rajshahi Rajshahi Bangladesh

**Keywords:** delamanid, drug‐resistance, molecular mechanism, treatment efficacy, tuberculosis

## Abstract

**Background and Aims:**

Tuberculosis (TB) remains a major global health problem, and treatment progress is increasingly threatened by rising multidrug‐resistant tuberculosis (MDR‐TB). Delamanid (DLM), a nitroimidazole drug, has shown good efficacy and safety against both drug‐susceptible and drug‐resistant *Mycobacterium tuberculosis* (*Mtb*) strains. However, data on its resistance mechanisms, drug susceptibility testing (DST), clinical effectiveness, safety, and pharmacokinetics remain limited. This review aims to summarize the most recent molecular, structural, and clinical evidence related to DLM.

**Methods:**

A comprehensive literature search was performed using WHO publications and major scientific databases, including PubMed, Web of Science, Embase, Scopus, and the Cochrane Library. Studies published through 2024 and early 2025 on DLM resistance, mechanisms of action, DST, pharmacokinetics, safety, and treatment outcomes were included. Structural analyses of key proteins involved in DLM activation were carried out using crystal structures and AlphaFold models.

**Results:**

Recent research identified multiple mutations in the F420‐dependent activation pathway, particularly in *ddn*, *fgd1*, *fbiA*, *fbiB*, *fbiC*, and *fbiD* that contribute to DLM resistance. Structural modeling demonstrated how these mutations affect protein stability and cofactor binding. Clinical studies showed that DLM‐containing regimens improve culture conversion and treatment success, especially when combined with oral agents such as bedaquiline and linezolid. Safety data indicate that DLM is generally well tolerated, with QT prolongation being the main but manageable adverse effect.

**Conclusion:**

DLM is an important and effective component of MDR‐/XDR‐TB treatment. A clearer understanding of its resistance mechanisms, pharmacological properties, and clinical outcomes can support better regimen design and help prevent the development of further resistance.

**Abbreviations**
BDQ: bedaquiline
BMD: broth microdilution method
CFZ: clofazimine
Ddn: deazaflavin‐dependent nitroreductase
Del: deletion
DLM: delamanid
DR: drug‐resistant
DR‐TB: drug‐resistant tuberculosis
DS: drug‐susceptible
DST: drug susceptibility testing
EMA: European Medicines Agency
EPPG: enoylpyruvoyl‐2‐diphospho‐5′‐guanosine
FQ+: fuoroquinolone resistance
Fs: frameshift
Ins: insertion
LZD: linezolid
MABA: microplate alamar blue assay
MDR‐TB: multidrug‐resistant tuberculosis
MGIT: mycobacteria growth indicator tube
*Mtb*
: *Mycobacterium tuberculosis*
NM: not mentioned
NTM: non‐tuberculous mycobacteria
OBR: optimized background regimen
PMD: pretomanid
Pre‐XDR: pre‐extensively drug‐resistant
RCSB PDB: Research Collaboratory for Structural Bioinformatics Protein Data Bank
REMA: resazurin microtiter assay
RGM: rapidly growing mycobacteria
RR: rifampicin‐resistant
SGM: slowly growing mycobacteria
SLI+: second‐line injectable resistance
TB: tuberculosis
WHO: World Health Organization
XDR‐TB: extensively drug‐resistant tuberculosis

## 1 Introduction

Tuberculosis (TB) is caused by the harmful organism *Mycobacterium tuberculosis* (*Mtb*) and is a serious global public health issue that claims over a million lives annually. Based on estimates, approximately 25% of people worldwide may have contracted TB [1]. Significantly, the global increase in TB incidence that began during the COVID‐19 epidemic has decreased and begun to stabilize. According to the World Health Organization (WHO) Global Tuberculosis Report 2024, an estimated 10.8 million people developed TB in 2023, with approximately 33% in women, 12% in children and young adolescents, and 55% in men. Although the 10.8 million cases in 2023 were greatly higher than the 10,400 thousand in 2021 and the 10,100 thousand in 2020, they were marginally higher than the 10,700 thousand in 2022. TB affects people of all age groups and is present in every country in some form. It is important to note that among the 1.25 million TB‐related deaths in 2023, approximately 0.161 million occurred in HIV‐positive individuals. This figure remains below the prepandemic estimate of 1.34 million deaths in 2019, as well as the projected 1.32, 1.42, and 1.40 million deaths for 2022, 2021, and 2020, respectively (https://www.who.int/teams/global-programme-on-tuberculosis-and-lung health/tb‐reports/global‐tuberculosis‐report‐2024). *Mtb* strains that are multidrug‐resistant (MDR), defined as *Mtb* strains resistant to isoniazid and rifampicin, pose a significant threat to the worldwide efforts to reduce TB. The WHO reported in 2023 that there were an expected 0.4 million new cases of rifampicin‐resistant (RR) and MDR TB worldwide [2]. The developmental stage of adult TB makes diagnosis and treatment especially challenging; thus, it is critical to comprehend the potential and problems associated with adult TB and with MDR/RR‐TB in particular. It is necessary to create precise and effective therapies as well as early diagnosis approaches to mitigate the impact of this disease. Treatment of MDR‐TB is a difficult task because there are few effective treatments available, and those that are can be costly, prolonged, or have side effects [3]. Globally, national TB programs reported to the WHO that, based on the cohort that commenced treatment in 2017, just 57% of those diagnosed with RR‐/MDR‐TB and 47% of those who were resistant to fluoroquinolones experienced positive treatment outcomes (https://www.who.int/publications/i/item/9789240013131). The rate of therapeutic success remains unacceptable, notwithstanding a minor increase in recent years. Significantly, research revealed that drugs used to treat MDR‐TB, like aminoglycosides, can have neurological adverse effects, hepatotoxicity, and irreversible ototoxicity. Thus, the discovery of novel anti‐TB drugs is a crucial approach for controlling the spread of TB, particularly in cases of TB that are drug‐resistant (DR). After 50 years of inaction in the search for novel TB targets and treatments, the new drug delamanid (DLM) has demonstrated encouraging efficacy in adult patients [4]. However, despite increasing global use of DLM since 2020, important gaps remain in understanding its molecular mechanisms of action and resistance, drug‐susceptibility testing parameters, pharmacokinetics, and updated clinical performance as of 2025. This gap limits optimal use of DLM within MDR‐TB regimens and underscores the need for an updated review focused specifically on its molecular basis of resistance and treatment efficacy.

**Figure 1:**
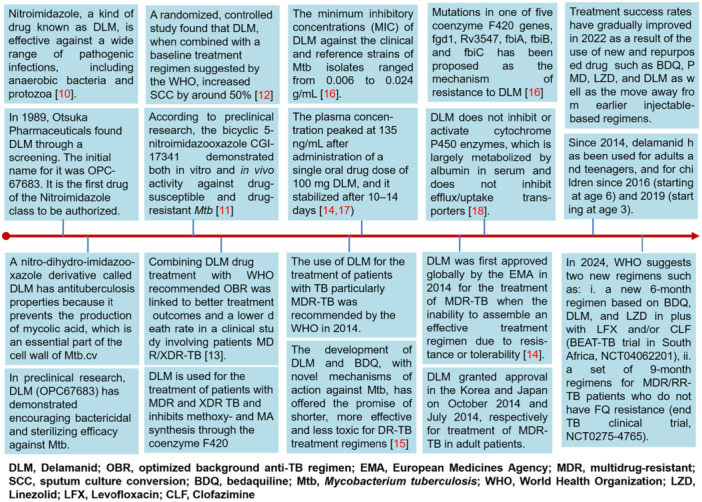
A brief update on the information for the development of the new drug delamanid.

The drug DLM, a nitroimidazole, has been suggested as part of new treatment plans for MDR–TB. DLM has emerged as a key component of MDR‐TB therapy as well as part of the shorter regimens. DLM was authorized by the European Medicines Agency (EMA) in 2014 as a new treatment for DR‐TB. It is a strong option for combination with other recently marketed anti‐TB drugs, like bedaquiline (BDQ), due to its tolerability and mode of action, which involves inhibition of mycolic acid formation [5]. DLM works against DR and drug‐susceptible (DS) strains of *Mtb*, notably MDR‐*Mtb* strains, by mainly preventing the formation of mycolic acid [6, 7]. According to the WHO report from 2020, the clinical studies of chemotherapy regimens containing DLM have progressed to phase III, and reports have been made regarding the clinical efficacy of both DLM and DLM‐containing regimens (https://www.who.int/publications/i/item/9789240013131). A brief update of information for the development of the new drug DLM is summarized in Figure 1 [8, 9, 10, 11, 12, 13, 14, 15, 16].

An increasing global concern is drug resistance [17, 18, 19]. Bacteria evolve drug resistance as a response to the challenges presented by therapeutic drugs. All specific pathogens are still sensitive to an antibiotic when it is first introduced from a therapeutic perspective; nevertheless, bacteria eventually develop resistance to the drug. Bacteria and antibiotics share an ecological niche, and microbes evolve defense mechanisms that fight off the damaging effects of drug compounds. Drugs have four primary targets in bacterial cells: protein synthesis, nucleic acid synthesis, the cell wall, and the membrane. Principal mechanisms behind drug resistance include inactivating a drug, altering a drug target, limiting drug uptake, and increasing active drug efflux [17]. The first step of developing drug use guidelines is to comprehend the mechanisms underlying the development of resistance.

Resistance to DLM has been associated with gene mutations, including *ddn, fbiA*, *fbiB*, *fbiC*, *fbiD*, and *fgd1*. DLM resistance could result from any alteration in one of the above genes. It is already known that resistance to DLM has been associated with mutations in *ddn* and *fgd1* [20]. Unfortunately, little is understood about the mechanisms underlying resistance, treatment efficacies, DST, and pharmacokinetics of DLM against the *Mtb* strain. Considering these concerns, we summarize and discuss in detail recent updates on DLM, including drug identification, mechanism of action, resistance, clinical efficacy, drug susceptibility, and pharmacokinetic profiles that have contributed to this drug being used in clinical or therapeutic settings.

Unlike earlier reviews that summarized only the basic mechanisms and clinical data available up to 2020–2022, this review incorporates the most recent findings from 2023 to early 2025, including newly identified mutations in *ddn, fbiA, fbiB, fbiC, fbiD*, and *fgd1*, along with a detailed structural analysis of how these mutations influence protein stability and F420/guanosine diphosphate (GDP) binding. Additionally, this review integrates updated evidence on DST, pharmacokinetics, safety, and real‐world treatment outcomes from newly published cohort studies in multiple countries. By combining molecular, structural, and clinical perspectives, this review provides the most comprehensive and up‐to‐date synthesis of DLM resistance and efficacy to date.

## 2 Methodology

The relevant information and data for our review manuscript were collected through an extensive search of WHO publications and various databases, including Web of Science, PubMed/MEDLINE, Embase, Scopus, and the Cochrane Library. We searched these sources to identify studies related to DLM against *Mtb* strains published throughout 2024 and early 2025. A combination of keywords, such as “TB,” “MDR‐TB,” “XDR‐TB,” “mechanism of action of DLM”, “resistance mechanisms of DLM,” “treatment efficacy of DLM,” “DST of DLM against *Mtb*,” “pharmacokinetics of DLM,” and “drug interactions,” was used to gather the information and data for this review manuscript. The review process adhered to the PRISMA (preferred reporting items for systematic reviews and meta‐analyses) 2020 guidelines, and the selection procedure was documented accordingly. A total of 2200 records were identified across all databases, of which 62 studies were included.

The structure files of Ddn, *FbiA*, *FbiB*, *FbiC*, *FbiD*, and *Fgd1* were retrieved from the RCSB Protein Data Bank and AlphaFold for structural analysis [21, 22]. The crystal structure of the *Mtb* Ddn core bound to F420 (PDB ID: 3R5R) was used to model additional Ddn mutations, while the AlphaFold structure AF‐P9WP15‐F1 was used for the R23P variant. The structures of *Mtb* Fgd1 (PDB ID: 3B4Y) and the C‐terminal region of FbiB bound to F420 (PDB ID: 4XOQ) served as templates for their respective mutants. SWISS‐MODEL was used to generate the wild‐type *M. smegmatis* FbiA structure using the FbiA–FO–GDP complex (PDB ID: 6UW5) as a template [23]. AlphaFold‐predicted structures of *Mtb* FbiC (AF‐P9WP77‐F1) and FbiD (AF‐A5WRN6‐F1) were used for modeling their variants. Free energy changes associated with point mutations were estimated using PremPS to assess effects on protein stability [24]. Protein–ligand interactions were visualized and analyzed using Discovery Studio Visualizer v4.5 and UCSF Chimera [25, 26].

### 2.1 Inclusion Criteria

1. Original research articles, reviews, clinical trials, case reports, or surveillance studies focused on DLM activity, mechanisms of action, resistance mechanisms, pharmacokinetics, drug interactions, or drug susceptibility testing (DST) against Mtb.
2. Studies published in English.
3. Publications appearing between January 2024 and February 2025.

### 2.2 Exclusion Criteria

1. Studies not specifically addressing DLM or its molecular targets.
2. Non‐peer‐reviewed materials (e.g., conference abstracts without full text, commentaries, editorials).
3. Duplicate records.
4. Studies lacking sufficient methodological detail or relevance to the scope of this review.

## 3 Results and Discussion

### 3.1 Molecular Mechanisms of DLM

#### 3.1.1 DLM

A new anti‐TB drug is DLM (OPC‐67683), a bicyclic nitroimidazole that exhibits strong TB action both in vivo and in vitro. Since 2014, DLM has been an important and effective anti‐TB drug that is used to treat DR and DS *Mtb* strains, including MDR‐TB [27]. DLM inhibits cell wall formation by blocking the biosynthesis of mycolic acid, which kills aerobically replicating *Mtb*. In anaerobic conditions, it causes respiratory toxicity through the emission of nitric oxide. The products of the *ddn*, *fbiA*, *fbiB*, *fbiC*, *fbiD*, and *fgd1* genes mediate the nitro‐reduction of the drug within the mycobacterial cell, which is necessary for these actions. DLM resistance has been linked to mutations in any of these six genes, at least in vitro and in mice. However, as of right now, little is understood regarding the prevalence and significance of the various genetic variations discovered in clinical settings. Notably, DLM exhibits partial cross‐resistance with pretomanid (PMD) and shares a similar activation pathway with PMD.

#### 3.1.2 Structure of DLM

**Figure 2:**
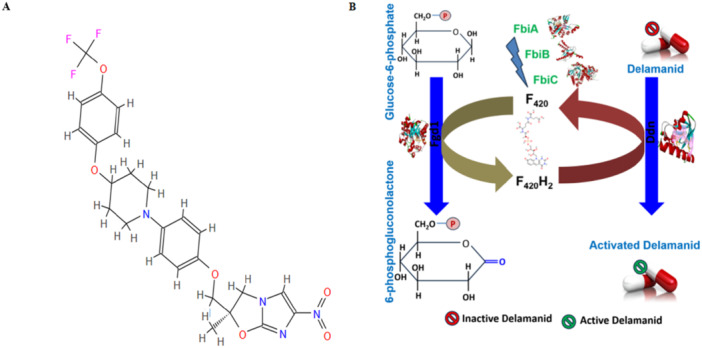
The chemical structure of delamanid (A) and how the drug is activated inside *Mycobacterium tuberculosis* (B). [Delamanid is a pro‐drug that requires the F420 cofactor system for activation. Fgd1 produces F420H₂, which donates electrons to Ddn. Activated delamanid then inhibits mycolic‐acid synthesis and generates reactive nitrogen species that damage the bacterial respiratory chain.].

DLM, also known as OPC‐67683, is formulated by Otsuka Pharmaceutical Co. to treat MDR‐TB and belongs to the bicyclic nitroimidazoles group with strong anti‐TB efficacy in vitro and in vivo against *Mtb* strains that are both DS and DR [28, 29, 30]. Over the past three decades, only two novel drugs have been invented as anti‐TB drugs, and DLM is one of them [31]. The chemical name of DLM is (2R)‐2‐Methyl‐6‐nitro‐2‐[(4‐{4‐[4(trifluoromethoxy)phenoxy]‐1‐piperidinyl} phenoxy) methyl]‐2,3‐dihydroimidazo[2,1‐b] [1, 3] oxazole. A class of molecules known as nitroimidazoles contains a five‐membered heterocycle in their structure, with a nitro group that can be found at positions 2, 4, or 5, and a pair of nitrogen atoms at positions 1 and 3 (Figure 2A). The nitroimidazole molecule, also referred to as 2‐nitroimidazole, is an analog of azomycin and a naturally occurring antibiotic derived from *Streptomyces eurocidicus* in the 1950s. The racemic mixture of 6‐nitro‐2,3‐dihydroimidazo[2,1‐b] oxazole acts on the *Mtb* complex through right‐handed agonists but not left‐handed ones.

#### 3.1.3 Mechanism of Action of DLM

In 2014, DLM was authorized by the EMA as a new treatment for DR‐TB [5]. Because of its mode of action (blocking the synthesis of mycolic acid) and its tolerability, it is an attractive option for combination with other recently approved anti‐TB drugs, such as BDQ [5]. By targeting various pathways in *Mtb*, this combination may improve results for DS‐ and DR‐TB and allow for the phase‐out of other second‐line anti‐TB drugs that are less effective and more toxic [32]. Two oral drugs that could be crucial for new, shorter regimens against DR‐TB are DLM and BDQ [33, 34]. The prodrug DLM needs to be bioactivated through the mycobacterial F420 coenzyme system, which contains the deazaflavin‐dependent nitroreductase Ddn (Rv3547), in order to have an antimycobacterial effect on *Mtb*. DLM has a dual bactericidal mechanism of action, that is, (1) disturbance with the synthesis of mycolic acid and (2) respiratory poisoning [35, 36].

#### 3.1.4 Disturbance With the Synthesis of Mycolic Acid

Mycolic acids are important and unique lipid components of the cell envelope of *Mtb* and related species, which are essential for growth and survival [37]. Enzymes that participate in the synthesis of mycolic acids are crucial for viability and virulence, making them new targets for drug discovery [36]. Three different mycolate species are produced in *Mtb*: α mycolates, which have two cis‐cyclopropane rings, and methoxy and keto series, which have a cis‐ or trans‐cyclopropane in the adjacent position along with the proximal oxygen function and an adjoining methyl branch [38]. Significantly, the synthetic process of ketomycolates and methoxymycolates is inhibited by DLM, but not that of α‐mycolates [28]. Methoxy‐ and ketomycolates are typically less abundant than α‐mycolates in *Mtb* and related organisms, though their precise ratios vary depending on growth conditions.

#### 3.1.5 Respiratory Poisoning

DLM, a prodrug, needs to be metabolically activated by the mycobacterial deazaflavin (F420)‐dependent nitroreductase (Ddn) (Figure 2B).

Of note, in vitro and clinical *Mtb* resistant isolates show mutations in the *ddn* gene or one of five other genes (*fgd1*, *fbiA*, *fbiB*, *fbiC*, *fbiD*) that are essential for prodrug activation. Like PMD, DLM is activated by the deazaflavin (F420) via the transfer of hydrides, generating unstable intermediates that ultimately result in the production of reactive nitrogen species such as nitrous acid and nitric oxide [39, 40]. Finally, the poisoning or interruption in the respiratory system is caused by reactive nitrogen species intermediates in the bicyclic nitroimidazole metabolic pathway [35, 39, 40, 41]. Of note, DLM exposure has been shown through transcriptional profiling of *Mtb* to possibly contribute to respiratory poisoning [35]. Recently, Hayashi et al. [42] found that DLM resistance can result from mutations in type II NADH dehydrogenase (ndh). According to this study, instead of nitric oxide, a NAD‐DLM adduct may be responsible for its anti‐TB activity.

### 3.2 Mechanism of Resistance to DLM

DLM, a relatively new anti‐TB drug that is part of Group C, is recommended for use in lengthier MDR‐TB regimens [43]. Known methods of resistance to DLM against *Mtb* strains are caused by mutations in the *ddn*, *fbiA*, *fbiB*, *fbiC*, *fbiD*, and *fgd1* genes, which are connected to the F420‐dependent bioactivation pathway, according to earlier studies [43, 44, 45]. The epidemiology of DLM resistance is summarized in Table 1 [43, 45, 46, 47, 48, 49]. In a recent study by Aghababa et al. [45], all 38 MDR‐TB isolates were sensitive to BDQ, while 7 (18.5%) of them showed resistance to DLM. According to an investigation of the sequencing data, the DLM‐resistant isolates had the most changes in the *ddn* gene, which included 18 nonsynonymous variants and 1 indel that resulted in frameshift mutations. Another study showed that 65 (65.00%) of the spontaneous DLM‐resistant strains had mutations in the known DR genes *ddn*, *fbiA*, *fbiB*, *fbiC*, *fbiD*, and *fgd1*, either through the F420 biosynthetic route (16.44% in *fbiA*, 5.48% in *fbiB*, and 21.92% in *fbiC*) or DLM prodrug activation (39.73% in *ddn*, 16.44% in *fgd1*) [43]. Significantly, 35% (35/100) of DLM‐resistant *Mtb* progeny strains had no known mutations in the *ddn*, *fbiA*, *fbiB*, *fbiC*, *fbiD*, and *fgd1* genes. A total of 45 mutations were identified, 38 of which had never been reported before. Among these 38 novel mutations, 3 were stop codon mutations, and 17 were frameshift mutations, both of which had a significant impact on protein structure. Nearly all of these mutations exhibited high levels of resistance to the DLM drug. The remaining 18 mutations were identified as point mutations, displaying varying levels of resistance to DLM. Similarly, Pang et al. [50] reported that 50% (2/4) of XDR‐*Mtb* clinical isolates had no known mutations in the six genes linked with DLM resistance. Another study from Iran demonstrated that 9/35 (25.7%) RR *Mtb* strains were resistant to DLM, including three RR, four MDR, and two pre‐XDR strains [51]. In contrast to the fact that this new compound (DLM) has not yet been adopted by Iranian TB patients as a treatment option, the high DLM resistance rate was astonishingly significant in the initial investigation. The R321S in the *fbiA* gene was the prevalent mutation found in 22.2% (2/9) DLM‐resistant strains. Notably, the Trp678Gly mutation in the *fbiC* gene was linked to a DLM‐susceptible clinical isolate [51]. A refractive index–based circular ring biosensor was developed to rapidly detect four types of *Mtb* cells, achieving high sensitivity (1500 nm/RIU) across the 2000–2050 nm wavelength range. It demonstrated excellent performance metrics, including a quality factor of 40,280, a figure of merit of 30,000, and a detection limit as low as 0.000005 for the TBC2 strain [52]. DLM and PMD share overlapping resistance mechanisms because both require activation through the F420‐dependent pathway, involving Ddn, Fgd1, and the F420‐biosynthetic genes (*fbiA*, *fbiB*, *fbiC*, *fbiD*). Mutations in any of these genes can disrupt prodrug activation and lead to cross‐resistance to both agents. However, this overlap is not complete; some *ddn* and *fbiC* mutations reduce susceptibility to DLM more than PMD, and vice versa. These differences reflect subtle variations in how each drug interacts with Ddn and F420. As shown in Table 2, we have summarized mutations in the genes *fgd1*, *ddn*, *fbiA*, *fbiB*, *fbiC*, and *fbiD* associated with resistance to DLM that have been identified by several recent studies [43, 45, 47, 49, 53, 54, 55, 56]. Subsequently, we analyzed point mutations, summarizing findings from various recent studies to investigate the impact of these mutations on protein structure in relation to DLM resis tance.

**Table 1:** Epidemiology of delamanid resistance.

References	Country	Published time	No. of Mtb isolates	No. of DLM‐resistant isolates	MIC (µg/mL)	Method for MIC	Investigating genes	Mechanism of resistance for mutation in genes	Resistance rate	Mutations rate	Study design
Aghababa et al. [45]	Iran	2024	38 MDR clinical strains	7	0.016 μg/mL	7H11 Middlebrook Agar	*ddn, fgd1*	*ddn, fgd1*	18.5%	57.1%	Observational (cross‐sectional)
The CRyPTIC Consortium [46]	23 different countries	2023	15,211 Mtb clinical strains	85	0.12 μg/mL	Broth microdilution plate assay	d*dn, fgd1, fbiA, fbiB, fbiC, fbiD*, Rv3249c	11 *ddn*, 7 *fbiA*, 1 *fbiC*	0.56%	22.35%	International cohort
Mok et al. [47]	Ireland	2023	900 Mtb clinical isolates	114	0.06 mg/L	BACTEC MGIT 960 system	*fgd1, ddn, fbiA, fbiB, fbiC*, Rv2983	*fgd1, ddn, fbiA, fbiB, fbiC*, Rv2983	114/900 (12.67%)	69/114 (60.53%)	Retrospective cohort
Liu et al. [43]	China	2022	60 (20 DS, 20 MDR, and 20 XDR clinical strains)	100	0.125	Resazurin microtiter assay (REMA)	*ddn, fgd1, fbiA, fbiB, fbiC, and fbiD*	*ddn, fgd1, fbiA, fbiB, fbiC*	—	65%	Experimental + clinical
He et al. [48]	11 provinces in China	2021	1603 Mtb clinical strains	11	0.12 μg/mL	UKMYC6 plates assay	*ddn, fbiA, fbiB, fbiC and fgd1*	No mutation any gene	0.7%	0	Retrospective surveillance
Wen et al. [49]	China	2019	220 (110 MDR‐ and 110 XDR‐TB strains)	7	0.2 mg/L	The microplate alamar blue assay (MABA)	*ddn, fbiA, fbiB, fbiC* and *fgd1*	*fbiA*	6.36%	14.29%	Observational

Abbreviations: DLM, delamanid; DS, drug‐susceptible; MDR, Multi‐drug resistant; Mtb; *Mycobacterium tuberculosis*; NM, not mentioned; NTM, non‐tuberculous mycobacteria; RGM, rapidly growing mycobacteria; SGM, slowly growing mycobacteria; XDR, extensively drug.

**Table 2:** Summary of recently identified mutations in ddn, fgd1, fbiA, fbiB, and fbiC associated with resistance to DLM in Mtb clinical, in vitro, and in vivo isolates.

Resistance genes	Nucleotide substitution	Amino acid change	DLM breakpoint	MIC method	S/R	Level of resistance	No. of isolates	Strains types	References
ddn	—	W20Stop	0.06 mg/L	Bactec MGIT 960 system	R	—	11	Clinical	Mansjö et al. [53] [51]
	—	Del G in aa 39	—	Broth Macrodilution assay	R	> 16 mg/L	1	In vivo mutants	Rifat et al. [54]
	68 G > C	R23P	0.125 μg/mL	REMA		0.25 μg/mL	1	In vitro mutants	Liu et al. [43]
	Del TAC at 86‐88	Y29del	0.06 mg/L	Bactec MGIT 960 system	R	> 0.24 mg/L	1	Clinical	Mok et al. [47]
	Del G at 92	R31fs	0.06 mg/L	Bactec MGIT 960 system	R	> 0.24 mg/L	1	Clinical	Mok et al. [47]
	113del	G38fs	0.125 μg/mL	REMA	R	16 μg/mL	1	In vitro mutants	Liu et al. [43]
	116_117insGb	G39fs	0.125 μg/mL	REMA	R	16 μg/mL	1	In vitro mutants	Liu et al. [43]
	117_118insC	T40fs	0.125 μg/mL	REMA	R	0.5‐1 μg/mL	2	In vitro mutants	Liu et al. [43]
	134del	P45fs	0.125 μg/mL	REMA	R	> 16 μg/mL	1	In vitro mutants	Liu et al. [43]
	ctg/cCg	L49P	0.12 μg/mL	UKMYC6 plate	R	—	1	Clinical	Zhao et al. [55]
	A151C	T51P	0.016 μg/mL	7H11 Middlebrook Agar	R	—	2	Clinical	Aghababa et al. [45]
	157 G > T	G53C	0.125 μg/mL	REMA	R	0.5 μg/mL	1	In vitro mutants	Liu et al. [43]
	161del	R54fs	0.125 μg/mL	REMA	R	16 μg/mL	1	In vitro mutants	Liu et al. [43]
	188 C > A	P63Q	0.125 μg/mL	REMA	R	> 16 μg/mL	2	In vitro mutants	Liu et al. [43]
	ctc/cCc	L64P	0.12 μg/mL	UKMYC6 plate	R	—		Clinical	Zhao et al. [55]
	195 C > G	Y65*	0.125 μg/mL	REMA	R	16 μg/mL	2	In vitro mutants	Liu et al. [43]
	G241A	G81S	0.016 μg/mL	7H11 Middlebrook Agar	R	—	4	Clinical	Aghababa et al. [45]
	gcc/Acc	A77T	0.12 μg/mL	UKMYC6 plate	R	—		Clinical	Zhao et al. [55]
	G229C	A77P	0.016 μg/mL	7H11 Middlebrook Agar	R	—	1	Clinical	Aghababa et al. [45]
	233 C > A	S78Y	0.125 μg/mL	REMA	R	> 16 μg/mL	1	In vitro mutants	Liu et al. [43]
	G241A	G81S	0.2 mg/L	BMD	R	0.4 mg/L	21	Clinical	Yang et al. [56]
	G241A	G81S	0.2 mg/L	BMD	R	0.8 mg/L	9	Clinical	Yang et al. [56]
	G241A	G81S	0.2 mg/L	BMD	R	> 1.6 mg/L	1	Clinical	Yang et al. [56]
	G242A	G81A	0.2 mg/L	BMD	R	> 1.6 mg/L	2	Clinical	Yang et al. [56]
	A266C	Y89S	0.016 μg/mL	7H11 Middlebrook Agar	R	—	1	Clinical	Aghababa et al. [45]
	A272C	D108A	0.016 μg/mL	7H11 Middlebrook Agar	R	—	1	Clinical	Aghababa et al. [45]
	A328C	T110P	0.016 μg/mL	7H11 Middlebrook Agar	R	—	1	Clinical	Aghababa et al. [45]
		R112W	—	Broth Macrodilution assay	R	> 16 mg/L	1	In vivo mutants	Rifat et al. [54]
	A343C	T115P	0.016 μg/mL	7H11 Middlebrook Agar	R	—	1	Clinical	Aghababa et al. [45]
	C348G	D116E	0.016 μg/mL	7H11 Middlebrook Agar	R	—	1	Clinical	Aghababa et al. [45]
	352 G > A	E118K		REMA	R	> 16 μg/mL		In vitro mutants	Liu et al. [43]
	A418C	T140P	0.016 μg/mL	7H11 Middlebrook Agar	R	—	2	Clinical	Aghababa et al. [45]
	A422C	D141A	0.016 μg/mL	7H11 Middlebrook Agar	R	—	1	Clinical	Aghababa et al. [45]
	434_435insC	P145fs	0.125 μg/mL	REMA	R	> 16 μg/mL	1	In vitro mutants	Liu et al. [43]
	447_448insC	E150fs	0.125 μg/mL	REMA	R	0.5‐16 μg/mL	3	In vitro mutants	Liu et al. [43]
	C447G	C149W	0.016 μg/mL	7H11 Middlebrook Agar	R	—	1	Clinical	Aghababa et al. [45]
	Del‐G at 92	Fs31^a^	0.016 μg/mL	7H11 Middlebrook Agar	R	—	1	Clinical	Aghababa et al. [45]
fgd1	—	K9N	—	Broth Macrodilution assay	R	0.5 μg/mL	1	In vivo mutants	Rifat et al. [54]
	209 T > G	L70R	0.125 μg/mL	REMA	R	16 μg/mL	4	In vitro mutants	Liu et al. [43]
	278 T > G	M93R	0.125 μg/mL	REMA	R	16 μg/mL	1	In vitro mutants	Liu et al. [43]
	603_604insG	L202fs	0.125 μg/mL	REMA	R	> 16 μg/mL	1	In vitro mutants	Liu et al. [43]
	851 G > C	W284S	0.125 μg/mL	REMA	R	> 16 μg/mL	1	In vitro mutants	Liu et al. [43]
	870del	P290fs	0.125 μg/mL	REMA	R	> 16 μg/mL	1	In vitro mutants	Liu et al. [43]
	911 G > T	G304V	0.125 μg/mL	REMA	R	> 16 μg/mL	1	In vitro mutants	Liu et al. [43]
	962 T > C	L321P	0.125 μg/mL	REMA	R	0.25 μg/mL		In vitro mutants	Liu et al. [43]
fbiA		G8A	0.06 mg/L	Bactec MGIT 960 system	R	0.24 mg/L	1	Clinical	Mok et al. [47]
	22 G > A	G8S	0.125 μg/mL	REMA	R	1 μg/mL	1	In vitro mutants	Liu et al. [43]
	—	Del G in aa 47	—	Broth Macrodilution assay	R	> 16 μg/mL	1	In vivo mutants	Rifat et al. [54]
	—	D49G	—	Broth Macrodilution assay	R	> 16 μg/mL	1	In vivo mutants	Rifat et al. [54]
	193 T > C	C65R	0.125 μg/mL	REMA	R	0.25 μg/mL	1	In vitro mutants	Liu et al. [43]
	309del	E103fs	0.125 μg/mL	REMA	R	16 μg/mL	1	In vitro mutants	Liu et al. [43]
	—	Q120P	—	Broth Macrodilution assay	R	> 16 μg/mL	1	In vivo mutants	Rifat et al. [54]
	428 T > C	L143P	0.125 μg/mL	REMA	R	> 16 μg/mL	1	In vitro mutants	Liu et al. [43]
	596 C > A	A199E	0.125 μg/mL	REMA	R	1 μg/mL	1	In vitro mutants	Liu et al. [43]
	—	S219G	—	Broth Macrodilution assay	R	0.03 μg/mL	1	In vivo mutants	Rifat et al. [54]
	745_809del	E249fs	0.125 μg/mL	REMA	R	> 16 μg/mL	1	In vitro mutants	Liu et al. [43]
	—	E249K	0.2 mg/L	MABA	R	> 16	1	Clinical	Wen et al. [49]
	747 A > G, 748 A > T	K250*	0.125 μg/mL	REMA	R	16 μg/mL	1	In vitro mutants	Liu et al. [43]
	—	D286A	—	Broth Macrodilution assay	R	> 16 μg/mL	1	In vivo mutants	Rifat et al. [54]
	864 G > A	W288*	0.125 μg/mL	REMA	R	> 16 μg/mL	1	In vitro mutants	Liu et al. [43]
	—	L308P	—	Broth Macrodilution assay	R	> 16 μg/mL	1	In vivo mutants	Rifat et al. [54]
fbiB	—	L15P	—	Broth Macrodilution assay	R	0.125 μg/mL	1	In vivo mutants	Rifat et al. [54]
	201del	P67fs	0.125 μg/mL	REMA	R	4 μg/mL	1	In vitro mutants	Liu et al. [43]
	—	L173P	—	Broth Macrodilution assay	R	0.125 μg/mL	1	In vivo mutants	Rifat et al. [54]
	735_736insC	A246fs	0.125 μg/mL	REMA	R	8 μg/mL	1	In vitro mutants	Liu et al. [43]
	991 A > T	I331L	0.125 μg/mL	REMA	R	16 μg/mL	1	In vitro mutants	Liu et al. [43]
	—	Del T in aa 684	—	Broth Macrodilution assay	R	0.125 μg/mL	1	In vivo mutants	Rifat et al. [54]
fbiC	—	‐C in aa 20	—	Broth Macrodilution assay	R	2 μg/mL	1	In vivo mutants	Rifat et al. [54]
	177 C > A	C59*	0.125 μg/mL	REMA	R	> 16 μg/mL	1	In vitro mutants	Liu et al. [43]
	343_344insC	R115fs	0.125 μg/mL	REMA	R	2‐16 μg/mL	6	In vitro mutants	Liu et al. [43]
	—	G194D	—	Broth Macrodilution assay	R	1 μg/mL	1	In vivo mutants	Rifat et al. [54]
	765del	F255fs	0.125 μg/mL	REMA	R	8 μg/mL	1	In vitro mutants	Liu et al. [43]
	773 G > A	G258D	0.125 μg/mL	REMA	R	> 16 μg/mL	1	In vitro mutants	Liu et al. [43]
	1130 T > G	L377R	0.125 μg/mL	REMA	R	8‐16 μg/mL	2	In vitro mutants	Liu et al. [43]
	—	L377P	—	Broth Macrodilution assay	R	> 16 μg/mL	1	In vivo mutants	Rifat et al. [54]
	—	C562W	—	Broth Macrodilution assay	R	> 16 μg/mL	1	In vivo mutants	Rifat et al. [54]
	1792 G > C	E598Q	0.125 μg/mL	REMA	R	16 μg/mL	1	In vitro mutants	Liu et al. [43]
	1810G > T	G604C	0.125 μg/mL	REMA	R	16 μg/mL	1	In vitro mutants	Liu et al. [43]
	2003_2007del	A668fs	0.125 μg/mL	REMA	R	> 16 μg/mL	1	In vitro mutants	Liu et al. [43]
	—	K684T	—	Broth Macrodilution assay	R	> 16 μg/mL	1	In vivo mutants	Rifat et al. [54]
	2092 C > G	H698D	0.125 μg/mL	REMA	R	> 16 μg/mL	1	In vitro mutants	Liu et al. [43]
	—	A827G	—	Broth Macrodilution assay	R	> 16 μg/mL	1	In vivo mutants	Rifat et al. [54]
	ggt/ggC	G839A	0.12 μg/mL	UKMYC6 plate	R	—	1	Clinical	Zhao et al. [55]

Abbreviations: aa, amino acid; BMD, broth microdilution method; Del, deletion; Fs, frameshift; Ins, insertion; MABA, the microplate alamar blue assay; NC, no change; R, Resistance; REMA, Resazurin microtiter assay; S, Susceptible; *, Stop codon.

### 3.3 Impact of Point Mutations on DLM Resistance (Structure Stability and Ligand Binding)

**Figure 3:**
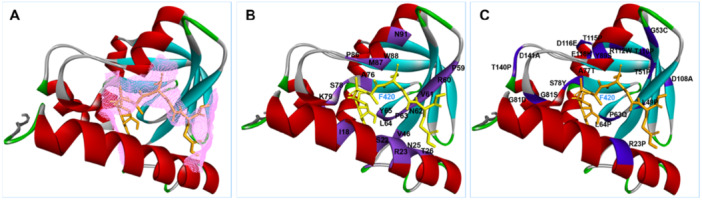
The 3D structure of the Ddn enzyme bound to its cofactor F420. Important amino acid residues surrounding the F420 binding pocket are highlighted, along with mutations known to cause delamanid resistance. These mutations often reduce F420 binding or disrupt enzyme stability (A–C).

During the DLM activation process, the Ddn protein utilizes the cofactor F420‐H_2_ to catalyze the reduction of nitroimidazole prodrugs, resulting in the intracellular release of reactive nitrogen species. Consequently, we conducted a structural analysis of the Ddn protein using the Ddn‐F420 complex (PDB ID: 3R5R) as a template (Figure 3A–C). As shown, residues I18, S22, R23, N25, T26, V46, P59, R60, V61, N62, P63, L64, Y65, A76, S78, K79, P86, M87, W88, and N91 are located near the binding site of F420. Notably, the mutations R23P, T51P, G53C, P63Q, L64P, and S78Y are in close proximity to the F420 binding site (Figure 3A–C).

**Figure 4:**
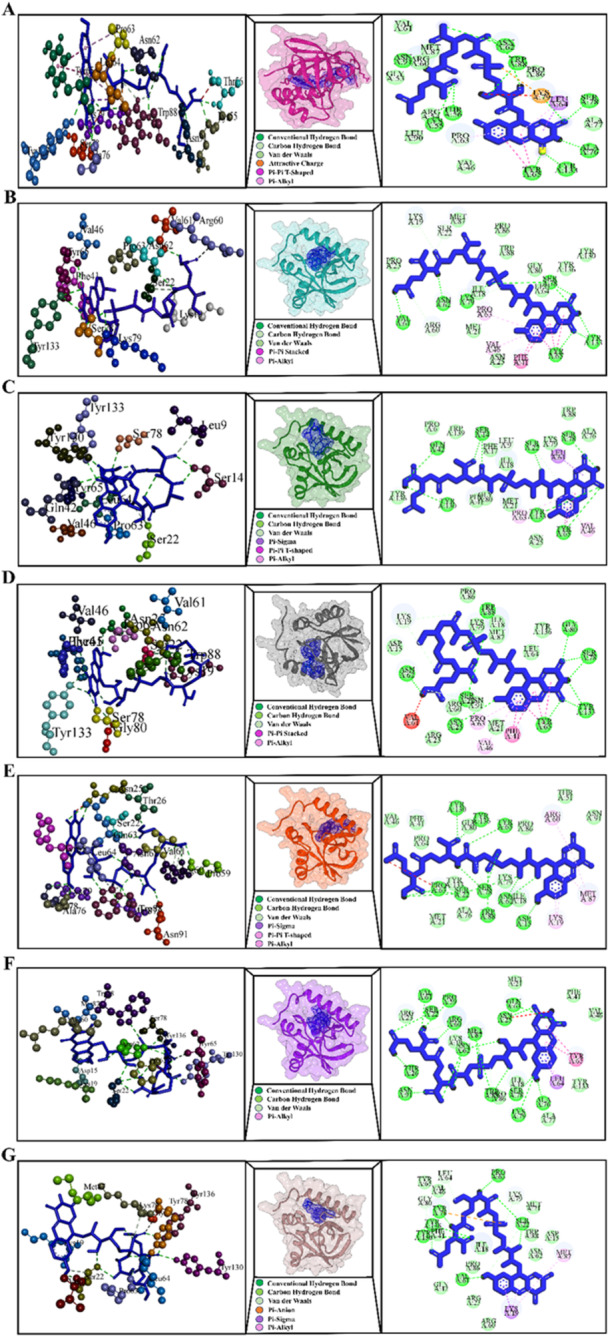
The 3D and 2D diagrams displaying the interactions between Ddn protein and cofactor F420: (A) wild type Ddn, (B) mutant R23P, (C) mutant T51P, (D) mutant G53C, (E) mutant P63Q, (F) mutant L64P, and (G) mutant S78Y. The F420 molecule (blue color) is shown in the middle of the 3D and 2D diagrams using a stick representation. These figures compare how F420 interacts with normal Ddn and clinically important mutants. Mutations weaken hydrogen bonding and alter the structure of the binding site, reducing DLM activation.

According to a thorough analysis of the interaction between Ddn and F420, the following mutations prevent receptor‐ligand binding: R23P, T51P, G53C, P63Q, L64P, and S78Y. Interestingly, compared to the wild type, these mutations (R23P, T51P, G53C, P63Q, L64P, and S78Y) have a greater impact on catalytic activity and show a less pronounced hydrogen bonding pattern (Figure 4A–G).

Furthermore, the mutations G53C, P63Q, and S78Y had a more significant effect on Ddn protein stability than R23P, as demonstrated by the lower DLM MIC value. The Eris method indicated that the T51P, G53C, P63Q, and L64P mutations in Ddn have a more substantial impact on protein stability than R23P and S78Y, as shown by the low ∆∆G values (Table S1). In contrast, the mutations L49P, A77T, A77P, G81S, G81D, Y89S, D108A, T110P, R112W, T115P, D116E, E118K, T140P, and D141A are located more than 5 Å from the F420 binding site (Figure 3B). Significantly, the calculated free energy (∆∆G) for L49P, A77T, A77P, G81S, G81D, Y89S, T110P, R112W, T115P, E118K, and T140P was higher, suggesting that these mutations alter drug resistance by modifying the stability of the protein structure and its interactions rather than by affecting cofactor F420 binding. The ∆∆G values for D108A, D116E, and D141A were low, and these mutations are far from the F420 binding site, indicating that they do not induce unfavorable interactions. Therefore, the impact of these mutations (D108A, D116E, and D141A) on drug resistance remains unclear, warranting further investigation to elucidate their role in DLM drug resistance.

**Figure 5:**
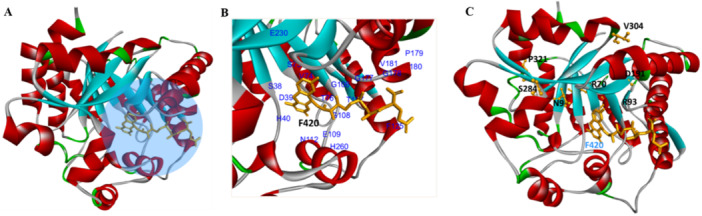
This figure shows the overall structure of Fgd1 bound to F420. Resistance‐related mutations are displayed, most of which lie outside the binding pocket and likely contribute to resistance by destabilizing the protein (A–C).

Mycobacteria contain Fgd1, which is involved in energy metabolism. F420‐dependent Fgd1 (glucose‐6‐phosphate [G6P] dehydrogenase) in *Mtb* uses the oxidized F420 cofactor, which is reduced to F420H2 for the activation of DLM, to catalyze the process that converts G6P to 6‐phosphogluconolactone. In this review, we have summarized mutations (K9N, L70R, M93R, G191D, W284S, G304V, and L321P) in the *fgd1* gene associated with DLM resistance that have been identified by several recent studies [43, 54]. Consequently, we conducted a structural analysis of the Fgd1 protein using the Fgd1‐F420 complex (PDB ID: 3B4Y) as a template (Figure 5). As shown, residues S38, D39, H40, S73, V74, T76, T107, G108, E109, N112, G177, G178, P179, A180, V181, E230, and H260 are located near the F420 binding site. According to a study by Liu et al. [43], the mutations L70R, M93R, G191D, W284S, G304V, and L321P in Fgd1 are positioned more than 8 Å from the F420 binding site. As a result, these mutations are likely to influence drug resistance by affecting structural stability rather than direct cofactor binding (Figure 5A–C). The K9N mutation in Fgd1, located approximately 5 Å from the F420 binding site, was found to have a high calculated free energy change (∆∆G). Additionally, this mutation introduced unfavorable interactions (Table S1). Considering these findings, the K9N mutation may contribute to drug resistance by destabilizing the protein structure rather than interfering with cofactor binding.

**Figure 6:**
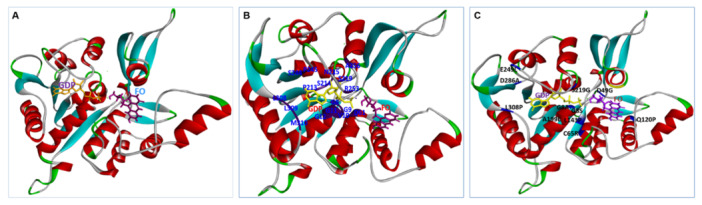
FbiA interacts with GDP and FO during F420 biosynthesis. Mutations near the substrate‐binding region reduce cofactor formation and contribute to delamanid resistance (A–C). GDP, guanosine diphosphate.

It is now known that a transferase encoded by FbiA catalyzes the formation of dehydro‐F420 by converting a phosphoenolpyruvyl moiety to 7,8‐didemethyl‐8‐hydroxy‐5‐deazariboflavin (FO). The FbiA protein utilizes enoylpyruvoyl‐2‐diphospho‐5’‐guanosine and FO as substrates to facilitate the biosynthesis of the redox cofactor F420. To model the crystal structure of M. smegmatis FbiA in complex with GDP and FO, we used the available structure (PDB ID: 6UW5) as a template to predict the FbiA‐FO‐GDP complex (Figure 6A–C). As summarized in Table 2, *we* identified several nonsynonymous mutations—G8A, G8S, D49G, C65R, Q120P, L143P, A199E, S219G, E249K, D286A, and L308P—that have been associated with DLM resistance in Mtb, based on findings from different studies [43, 47, 49, 54]. Therefore, only mutations G8A, G8S, S219G, and L308P were situated close to the GDP substrate binding site among our detected mutations in FbiA from several studies (Figure 6A–C).

**Figure 7:**
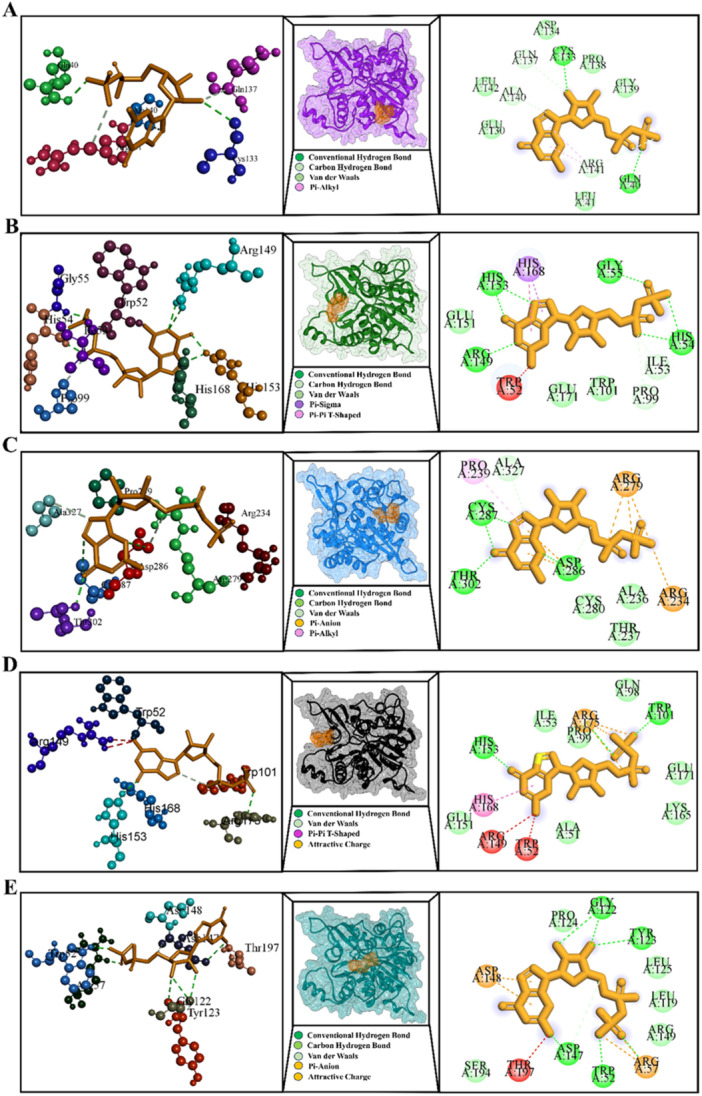
The 3D and 2D diagrams illustrate the interactions between the FbiA protein and GDP: (A) wild‐type FbiA, (B) mutant G8A, (C) mutant G8S, (D) mutant S219G, and (E) mutant L308P. This figure compares GDP binding between normal FbiA and resistant mutants. Altered bonding and structural distortions weaken GDP association and impair F420 synthesis. GDP, guanosine diphosphate.

Mutations in G8A, G8S, S219G, and L308P caused barriers to receptor‐ligand interaction, according to our comprehensive *in silico* analysis of the interaction between FbiA and GDP. Remarkably, the Gly8Ala, G8S, S219G, and L308P mutations affect catalytic activity more than the wild type, and their hydrogen bonding pattern is less noticeable (Figure 7A–E). Other mutations, D49G, C65R, Q120P, L143P, A199E, Glu249Lys, and D286A, are located far away, 5 Å from the GDP binding site, and their computed ΔΔG values were extremely positive, indicating that this mutation may have an allosteric effect on reducing the stability of the FbiA‐GDP complex (Table S1).

**Figure 8:**
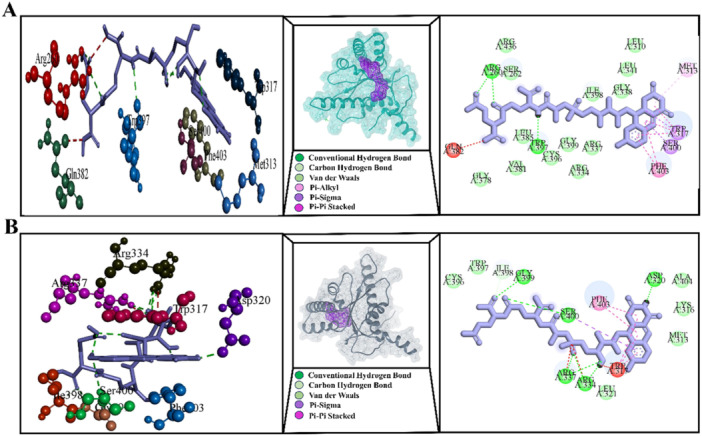
The 3D and 2D diagrams depict the interactions between the FbiB protein and the cofactor F420 for (A) wild‐type FbiB and (B) mutant I331L.

The coenzyme F420 plays a crucial role in various biological processes, including methanogenesis in archaea, prodrug activation in *Mtb*, and antibiotic production in *streptomycetes*. Its biosynthesis is catalyzed by *FbiB*, which utilizes glutamate and dehydro‐F420 as substrates. The crystal structure of the F420‐bound *Mtb*‐FbiB C‐terminal domain (PDB ID: 4XOQ) is shown in Figure S1A. As depicted in Figure S1B, residues H291, P354, D355, G356, A357, S359, Y360, T366, E369, and H370 are positioned near the F420 binding site. Several FbiB mutations—L15P, L173P, I331L, and W397R—have been reported in different studies, with I331L being the only mutation located close to the F420 binding site (Figure S1C). The AlphaFold‐predicted structure (AF‐P9WP79‐F1) suggests that L15 is part of a β‐strand at the N‐terminus of FbiB, and the L15P mutation (associated with a DLM MIC of 0.125 μg/mL) likely disrupts this β‐strand, leading to structural instability. The DLM MIC of 0.125 μg/mL is linked to the L173P mutation, which is situated in a coil area and is far from the F420 binding site. Its calculated ΔΔG values were extremely positive, indicating that it might have an allosteric impact and lessen the FbiB‐F420‐H2 complex's stability (Table S1). Notably, the I331L mutation caused undesirable bumps with the ligand F420 in addition to the residues R334 and R337 of the *Mtb* FbiB protein (Figure 8A,B).

**Figure 9:**
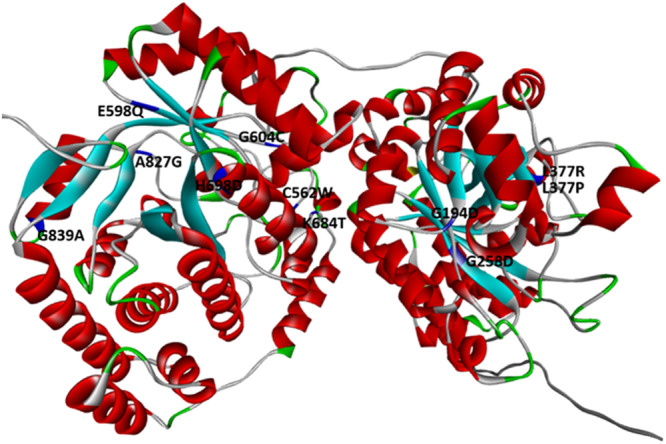
The structure of FbiC in Mtb, along with the identified mutated residues (black), is depicted using a blue‐ribbon representation.

The FbiC protein catalyzes the radical‐mediated synthesis of FO. Since no crystal structure for FbiC was available, structural analysis was performed using the AlphaFold‐predicted model (AF‐P9WP77‐F1) (Figure 9).

Free energy calculations (Table S1) suggest that mutations G194D, G258D, L377R, L377P, C562W, E598Q, G604C, K684T, H698D, A827G, and G839A may result in structural instability. Therefore, we focused on analyzing the potential interactions that impact the stability of the structure. Liu et al. [49] reported that mutations G258D, L377R, and G604C introduced unfavorable bumps. The calculated ∆∆G energy for G194D, L377P, C562W, E598Q, K684T, H698D, A827G, and G839A in FbiC was high, and these mutations introduced unfavorable interactions. Taking into account that these alterations may change resistance to drugs by modifying structural stability.

### 3.4 DST and DLM

Standardized and repeatable procedures for *in vitro* DST should be established concurrently with the release of novel antimicrobials to halt the early development of drug resistance and the transmission of resistant strains. Schena et al. [57] reported that spontaneous resistance to DLM underscores the need for DST before its clinical use in treatment regimens. This precaution helps prevent the selection and spread of DLM‐resistant strains, a pattern observed with other anti‐TB drugs. Various DST techniques are available for DLM, with liquid media‐based methods providing results in under 10 days, around 3 weeks quicker than the proportional approach based on agar [57]. This accelerated detection enables earlier treatment adjustments and helps curb the spread of DR‐TB. Both the REMA and Mycobacteria growth indicator tube (MGIT) methods effectively and accurately differentiate between *Mtb* strains susceptible or resistant to DLM, using a breakpoint concentration of 0.125 mg/L.

### 3.5 Treatment Efficacies of DLM

Treating DR‐TB poses significant challenges for physicians and national TB programs due to the limited availability of effective drugs and inexperience with more recent regimens. Managing MDR‐ and XDR‐TB is particularly difficult, as treatment options are scarce, costly, and require prolonged therapy, often accompanied by severe side effects. Research has shown that drugs used for MDR‐TB, such as aminoglycosides, can cause neurological complications, hepatotoxicity, and irreversible ototoxicity [29].

The novel drug DLM (OPC‐67683), a member of the nitro‐dihydro‐imidazooxazole class, prevents mycolic acid production. It has shown potent early bactericidal activity in clinical trials and strong preclinical efficacy, both in vivo and in vitro, against DR and DS *Mtb* strains [58]. After decades of stagnant research on new TB treatments, the recently developed drugs BDQ and DLM have shown promising success in treating TB in adults [4]. Research indicates that adding BDQ or DLM to an optimized background regimen (OBR) improves treatment outcomes and reduces mortality risk. Additionally, antibacterial drugs like clofazimine (CFZ) and linezolid (LZD), known for their antimycobacterial properties, are being repurposed for DR‐TB treatment [59, 60]. A few additional antibacterial drugs, such as CFZ and LZD, have anti‐mycobacterial features and are being repurposed for the treatment of DR‐TB.

DLM, BDQ, and PMD are the key new oral agents for MDR‐/XDR‐TB therapy. All three improve culture conversion and treatment success, with BDQ showing strong bactericidal activity and PMD contributing to high cure rates in BPaL/BPaLM regimens. DLM provides significant early bactericidal activity and enhances outcomes in longer MDR‐TB regimens. Resistance differs among the drugs: BDQ resistance is rising in some settings, while DLM and PMD share F420‐dependent activation, leading to overlapping resistance mutations. Safety profiles vary BDQ and DLM may prolong QTc, whereas PMD is generally well tolerated but depends on companion‐drug toxicity. These agents are complementary, and their selection should be guided by susceptibility testing and safety considerations.

Rather than taking each drug alone with OBR, country programs should think about efficiently controlling DR‐TB by mixing new drugs with repurposed drugs [61]. After 6 months of treatment, treatment regimens combining BDQ and DLM showed adequate culture conversion with no additive or synergistic QTc‐prolongation [62, 63]. The WHO has also regrouped anti‐TB drugs and advised combining drugs from groups A and B such that a regimen contains at least four effective drugs [64]. As shown in Table 3, studies on DLM‐containing regimens conducted in various countries, including Nigeria, China, India, and Congo, have reported high treatment success rates ranging from 70% to 91% [64, 65, 66, 67].

**Table 3:** Summary of observational research studies describing for DR‐TB/RR‐TB/Pre‐XDR‐TB patients treated with DLM‐containing individual or standardized shorter/longer treatment regimen others drug.

Author	Study design	Country	Year	Mean age	HIV	Clinical strains	No. of TB patients receiving DLM	Drugs used in the regimen	Treatment duration	Outcomes		
										Treatment success	Failure	Death
Fadeyi et al. [65]	Prospective single‐arm	Nigeria	2024	35	5% (1/20)	DR‐TB/RR‐TB/Pre‐XDR‐TB	20	DLM, BDQ, CFZ, LZD	9 months	70% (14/20)	0	25% (5/20)
Chen et al. [66]	Retrospective cohort	China	2024	37	0	MDR/RR/pre‐XDR‐TB	25	WHO‐recommended and China	22 weeks	84% (21/25)	0	0
Padmapriyadarsini et al. [64]	Prospective cohort	India	2023	27	NR	MDR‐TBFQ + /MDR‐TBSLI+	165	DLM, BDQ, CFZ, LZD	24–36 weeks	91% (139/153)	1.3% (2/153)	2.6% (4/153)
Kashongwe et al. [67]	Retrospective study	Congo	2020	35.83	8.3% (1/12)	MDR‐TB/pre‐XDRTB	12	DLM, Bdq, Km, Pas, Lzd, H, Cfz, Z, Lfx, Mpm‐Clv, Am, Ipm‐clv, Cs	20 months	83.3% (10/12)	0	8.3% (1/12)

Abbreviations: Am, amikacin; BDQ, bedaquiline; CFZ, clofazimine; Cs: Cycloserine; DLM, delamanid; FLQ, fluoroquinolone; H, Isoniazid; H: Isoniazid; Ipm‐clv, Imipenem‐clavulanate acid; Km, Kanamycin; Lfx, Levofloxacin; LZD, linezolid; MDR, multidrug‐resistant; MDR‐TBFQ + , MDR‐TB with additional resistance to fluoroquinolones; MDR‐TBSLI + , second‐line injectable; Mpm‐Clv, Meropenem‐clavulanate; NR, not reported; PAS, para‐amino salicylic acid; Pre‐XDR, Pre‐extensively drug‐resistant; RR, rifampin‐resistant; XDR, extensively drug‐resistant; Z, Pyrazinamide.

According to a recent systematic review and meta‐analysis study, patients with MDR‐TB receiving DLM had high rates of culture conversion and treatment success (72.5%; 95% CI: 44.2–89.8 and *p* < 0.001) despite widespread resistance and few adverse effects [68]. Another significant study from India found that patients with fluoroquinolone resistance (MDR‐TBFQ+) or second‐line injectable resistance (MDR‐TBSLI+) had a 91% favorable outcome when using DLM and BDQ in combination with other drugs in a short‐term, completely oral regimen, and 69% when treated with resistance to both FQ and SLI [64].

When combined with moxifloxacin, CFZ, or pyrazinamide, the BDQ‐DLM regimen demonstrated more favorable outcomes than those observed in a South African cohort study [61], although it was comparable to the EndTB observational study [69]. Even though the majority of the patients in the cohort trial had significant bacterial loads in their sputum and substantial bilateral illness, they were still able to obtain higher culture conversion at the end of the treatment period [64]. This finding is probably due to the favorable treatment response observed when BDQ and DLM were combined with repurposed drugs. The outcomes of this study are likewise similar to the NixTB experiment, which included PMD, LZD, and BDQ in combination and had a 90% success rate [70].

A noteworthy study by Skripconoka et al. [11] shows that when DLM is used for at least 6 months combined with an OBR that is advised by the WHO, a greater percentage of patients obtain favorable outcomes and a lower death rate. Compared to 126 (55%) of 229 patients who had DLM for at least 2 months, 143 (74.5%) of the 192 patients who received it for at least 6 months experienced it. Similarly, among the subset of patients with XDR‐TB, a higher percentage observed favorable results and a decreased death rate after receiving DLM for at least 6 months. Interestingly, after using DLM for at least 6 months, all 44 XDR‐TB patients survived [11]. According to this investigation, individuals with both MDR and XDR TB may benefit from treatment with DLM for 6 months when combined with an OBR, and this approach can also lower mortality rates.

Treatment regimens containing DLM have demonstrated increased efficacy in the management of XDR‐TB and MDR‐TB. However, despite relatively low rates, treatment failure and mortality remain concerning and should not be disregarded. One significant factor contributing to these adverse outcomes is inadequate adherence to treatment. For optimal therapeutic efficacy, patients must complete the full course of DLM. A higher risk of treatment failure and death is linked to nonadherence to the recommended regimen. Suboptimal drug consumption may also contribute to the development of resistance in the population. Evidence consistently supports that DLM is a potent anti‐TB agent that improves culture conversion rates, which correlates with favorable treatment outcomes. A short course, fully oral regimen containing DLM has shown efficacy in curing MDR‐TB and fluoroquinolone‐resistant MDR‐TB (MDR‐TBFQ+). Additionally, DLM has been shown to be safe for prolonged use. For MDR‐TB patients who exhibit inadequate response to standard therapies or have limited treatment options, DLM should be considered as a valuable therapeutic option.

### 3.6 Drug Interactions

The anti‐TB drug DLM, which belongs to the nitro‐dihydro‐imidazole class of compounds, prevents the *Mtb* cell wall from synthesizing mycolic acid. Significantly, DLM showed strong in vitro and in vivo activity against DR and DS strains of *Mtb* during preclinical development [28]. DLM demonstrated significant efficacy in early bactericidal studies during clinical development in patients with DS TB [58]. Compared to treatment with a control group with an OBR, treatment with DLM for 2 months dramatically increased 2‐month sputum culture conversion by roughly 50% in MDR‐TB patients [10]. Furthermore, a longer‐term observational trial found that treatment with DLM plus an improved background regimen for at least 6 months was linked to considerably decreased mortality and higher favorable treatment outcomes (74.5% vs. 55%) when compared to treatment for at least 2 months [11]. DLM received authorization in 2014 for the treatment of pulmonary multidrug‐resistant tuberculosis (MDR‐TB) in adult patients in the European Union, Japan, and the Republic of Korea based on these findings. Adults are currently advised to take 100 mg twice daily of DLM with food [71].

DLM drug is mostly metabolized by albumin to DM‐6705; cytochrome P450 (CYP)‐mediated pathways are thought to be involved in the metabolism of DM‐6705 to additional metabolites [72]. Albumin is a unique part of the major drug metabolism route, despite the fact that some drugs are partially metabolized by it. DLM did not appear to have any clinically significant drug‐drug interactions with CYP isoenzyme inducers or inhibitors, but it was not possible to rule out interactions resulting from some degree of CYP involvement. In line with the in vitro data, the drug‐drug interaction investigations involving DLM demonstrate little effect on DLM absorption because of CYP enzyme activation or inhibition [73]. The metabolism of DLM is not greatly affected by CYP3A4, according to the outcomes of investigations using CYP3A4 inducers (efavirenz and rifampin) or inhibitors (ritonavir). This conclusion is consistent with the in vitro data. Furthermore, coadministering DLM with other drugs whose metabolism is mediated by CYP450 pathways is safe, as there is little chance of clinically important CYP‐related drug‐drug interactions [73]. The studies concluded DLM is a promising way to reduce drug–drug interactions since it is primarily digested by albumin rather than CYP enzymes [74]. With antiretroviral drugs, there are no known drug–drug interactions [73]. In addition, the concomitant use of DLM does not appear to pose any new safety issues, according to a recent data assessment conducted for the WHO recommendations [75].

### 3.7 Safety and Tolerability of DLM

The MDR/RR‐TB treatment has a terrible track record due to its long duration, high costs, high toxicity, large pill burden, little efficacy, serious adverse events (AEs), and high mortality rates [76, 77]. Even though clinicians are aware of the AEs that patients experiencing various combinations of drugs regularly suffer, they are typically poorly reported and mistakenly believed to be unavoidable. A number of drugs have been in use for many years, including injectables (amikacin, kanamycin, and capreomycin), prothionamide/ethionamide, terizidone/cycloserine, and para‐aminosalicylic acid (PAS); however, patients have rarely been given options, even though it is known that they can suffer AEs. However, the approach to treating MDR/RR‐TB has changed for patients as well as physicians due to the rising usage of BDQ, DLM, and so‐called repurposed drugs like CFZ and LZD.

The incidence and frequency of clinically important AEs of special interest in MDR/RR‐TB patients on longer BDQ and/or DLM‐containing regimens were investigated in a very recent study by [78]. They discovered that AEs generally associated with injectable drugs and LZD were more prevalent than those commonly associated with BDQ and DLM. Similar findings by Huerga et al. [79] reported that using Bdq and Dlm concurrently with LZD and CFZ is safe and efficacious for patients with MDR/RR‐TB severe disease. It is a viable therapeutic option for people who are resistant to many anti‐TB drugs to use these drugs concurrently. A significant study reported that DLM was tolerated very well even in people who had used the drugs for more than 6 months. Another trial showed that DLM was well‐tolerated and had a very well‐defined safety profile [31]. A very recent study reported by Schönfeld et al. [80] found that even in patients receiving DLM for longer than 24 weeks, no additional safety issues were identified. Prolonged QT intervals were effectively treated and did not result in any cardiac complications that were clinically relevant. The results of the treatment met the WHO goal for Europe. It can be concluded that DLM has been proven to be safe, efficacious, and well‐tolerated in clinical trials involving adults with MDR/XDR‐TB who received 100 or 200 mg of the drug twice a day in addition to a standardized background therapy.

### 3.8 Pharmacokinetics of DLM

*Mtb* possesses the ability to tolerate anti‐TB drug resistance even when strong drugs are used in different combination therapy regimens [81]. A well‐designed pharmacological regimen can support clinical and bacteriological treatments while preventing the development and spread of resistance. The care of MDR‐TB and other chronic TB cases is clinically problematic due to limited treatment choices, which also raises concerns for public health. Similarly, inadequate administration of prescribed drugs during the intensive and continuation stages of therapy not only affects patient survival but also develops increased resistance. New drugs, such as DLM, have brought about a significant change in the treatment of TB. DLM was conditionally approved by the EMA as a first‐in‐class bicyclic nitroimidazole, based on the need to treat MDR‐TB and encouraging phase IIb study findings [30]. A prodrug called DLM is used to treat MDR/RR‐TB. It functions by activating the drug by decreasing the nitro group of the F420‐dependent nitroreductase.

The nitroimidazole molecule DLM is digested by albumin to produce DM‐6705. Although this metabolic route has been identified in vitro [15, 29], there has never been a clear connection established between the levels of plasma albumin in patients and the metabolism of DLM in humans [5]. The pharmacokinetic diversity observed in clinical practice is a result of multiple factors influencing the bioavailability of various drugs, such as drug–herb interactions, drug–food interactions, and CYP3A4 interactions. A recent study with 744 patients receiving DLM found that hypoalbuminemia appeared to improve DLM clearance [82]. This could be explained by the strong protein binding (> 99.5%) of DM‐6705 and DLM [15]. Thus, hypoalbuminemia may result in a reduction in protein binding and an increase in the overall clearance of DLM [82]. Other significant aspects of DLM pharmacokinetics include the fact that food has a significant impact on absorption (a meal has an approximate two‐fold increase in bioavailability when taken with food) [29] and that CYP enzymes are only necessary for the digestion of metabolites [15, 73].

Because DLM is not very soluble in water, the body may find it difficult to absorb. However, oral bioavailability varies from 35% to 60% and rises with meal intake, especially with high‐fat foods, according to animal studies [14]. According to Liu et al. [29], DLM has little chance of interfering with antiretroviral drugs and no interactions with CYP enzymes.

Moreover, DM‐6705 has a prolonged terminal half‐life of 121–322 h, whereas DLM has an elimination half‐life of 30–38 h [29]. Age, race, sex, or renal impairment do not appear to impact DLM pharmacokinetics [5]. It can be concluded that when DLM is administered with meals as compared to when it is taken without, its relative bioavailability increases. In addition, humans have an oral bioavailability of 25%–47% for DLM, which rises dramatically with a high‐fat diet. Along with this, DLM has a lengthy half‐life of 30–38 h due to its strong protein binding (99.5%).

### 3.9 Novelty, Limitations, and Future Perspectives

The current evidence on DLM offers important advancements in understanding its utility for MDR‐TB management. Notably, recent studies have elucidated the drug's molecular activation pathway via the F420‐dependent system, highlighting how mutations in ddn, *fgd1*, and *fbiA*/B/C/D directly impair prodrug activation. These mechanistic insights represent a significant step in predicting and diagnosing resistance, strengthening the rationale for integrating both phenotypic and targeted genotypic susceptibility testing into routine practice. Additionally, updated clinical data demonstrate meaningful improvements in treatment outcomes when DLM is administered for extended durations (≥ 6 months) as part of optimized combination regimens, with safety profiles remaining acceptable under appropriate QTc monitoring. This positions DLM as a valuable add‐on agent, especially for patients with limited effective drug options.

Despite these strengths, several limitations persist. Much of the clinical evidence supporting DLM effectiveness stems from observational cohorts rather than randomized controlled trials, which restricts the certainty of comparative efficacy. DST testing remains incomplete because a proportion of resistant isolates do not exhibit mutations in known F420‐pathway genes, indicating additional, undiscovered mechanisms of resistance. Pharmacokinetic variability, particularly in populations with hypoalbuminemia, pediatric patients, pregnant women, and individuals with hepatic impairment, remains insufficiently characterized. Moreover, the long‐term safety of DLM in combination with other QT‐prolonging agents requires continued vigilance, given the possibility of rare but clinically significant cardiac events.

Future research should focus on expanding rapid DST capacity using standardized phenotypic assays and targeted sequencing panels to improve early detection of resistance. Comparative clinical trials are needed to define the optimal role of DLM alongside newer regimen backbones, such as BDQ‐ and PMD‐based therapies. Further PK/PD studies in special populations are essential to refine dose optimization and monitoring strategies. Mechanistic investigations exploring non–F420‐dependent resistance pathways will fill current knowledge gaps and strengthen surveillance systems. Continued postmarketing safety monitoring and implementation research will also be critical to ensuring safe, effective, and programmatically feasible integration of DLM into global MDR‐TB treatment strategies.

## 4 Conclusions

DLM has been incorporated into treatment options for MDR‐ and XDR‐TB, offering an additional mechanism of action for managing DR Mtb. This article provides data on the effectiveness of DLM both alone and in combination with other drugs for the treatment of DR and MDR‐TB patients. For MDR‐TB patients, the addition of DLM to therapeutic regimens significantly improves outcomes, with high culture conversion rates and low mortality observed when DLM‐containing regimens are used.

Additionally, this review supports and details findings on drug resistance and DST for DLM in both in vitro and clinical *Mtb* strains. Drug resistance substantially reduces the effectiveness of treatment, highlighting the need for phenotypic and genotypic testing before treating MDR‐TB patients with DLM alone or in combination.

While DLM offers important therapeutic benefits, it should not be interpreted as a core MDR‐TB drug. WHO classifies DLM as a Group C drug, meaning it should be added only when the core Group A and B agents, such as BDQ, LZD, levofloxacin/moxifloxacin, and CFZ cannot construct a fully effective regimen. Therefore, the therapeutic impact of DLM in practice is dependent on its use within optimized combination regimens, rather than as a standalone contributor. This review summarizes the challenges surrounding the use of DLM against *Mtb* strains, which could assist in the development and discovery of new anti‐TB drugs and drug combinations. A thorough understanding of DST, pharmacokinetics, resistance‐associated mutations, and the therapeutic efficacy of DLM against *Mtb* can provide valuable insights into improving treatment outcomes, reducing mortality, preventing drug resistance, and halting the spread of TB. It will also facilitate the development of novel TB drugs and rapid molecular diagnostic methods.

## Author Contributions

**Md. Mahmudul Islam:** conceptualization, investigation, methodology, software, data curation, writing – original draft, resources. **Md. Zahid Hasan:** investigation, writing – original draft. **Md. Touki Tahamid Tusar:** writing – original draft, data curation. **Md. Yeamin Hossain:** investigation, validation, writing – original draft. **Md. Motaher Hossain:** writing – original draft, visualization, validation. **Md. Abdulla Al Jubayed:** Writing – original draft, investigation. **Md. Jubaer‐Al‐Abedin:** methodology, visualization, validation, writing – original draft, investigation. **Sheikh Soikot:** writing – original draft, software, visualization, validation. **Shanzida Akther:** writing – original draft, validation, software. **Jahid Bhuyian:** investigation, validation, formal analysis. **Hafizur Rahman Gazi:** writing – original draft, visualization, methodology. **B. M. Mahmudul Hasan:** writing – original draft, software. **Md Shofiul Azam:** writing – original draft, formal analysis, validation. **Md. Enamul Haque:** writing – original draft, formal analysis, visualization. **Abdullah‐Al‐Jubayer:** writing – original draft, methodology, validation. **Md. Faruk Hasan:** writing – original draft, software. **F. M. Ali Haydar:** writing – original draft, visualization. **Md. Khalekuzzaman:** writing – original draft, conceptualization. **Md. Torequl Islam:** writing – review and editing, visualization, validation. **Sohel Hasan:** supervision, project administration, writing – review and editing.

## Funding

The authors have nothing to report.

## Conflicts of Interest

The authors declare no conflicts of interest.

## Transparency Statement

The lead author Md. Torequl Islam, Sohel Hasan affirms that this manuscript is an honest, accurate, and transparent account of the study being reported; that no important aspects of the study have been omitted; and that any discrepancies from the study as planned (and, if relevant, registered) have been explained.

## Supporting information


**Figure S1:** Structure of FbiB of the Mtb complexed with F420 cofactors. (**A**) Ribbon representation of Mtb FbiB bound cofactor F420 (PDB ID: 4XOQ). The F420 (orange) is demonstrated with displaying style of stick. (**B**) Residues in FbiB are close to the cofactor F420 binding site. (**C**) A ball and stick display were used to represent the recently identified mutant residues (purple). The BIOVIA Discovery Studio Visualizer v.4.5 program was used to acquire these images.
**Table S1:** The effect of point mutations on the proteins (Ddn, Fgd1, FbiA, FbiB, and FbiC) stability and MIC range of mutants associated with DLM resistance against Mtb.

## Data Availability

The data that support the findings of this study are available from the corresponding author upon reasonable request. Data will be made available on request to the authors.
