# Supplementary material for: Molecular Mechanisms of Resistance and Treatment Efficacy of Delamanid Against Mycobacterium tuberculosis: A Systematic Review
Source: Health Sci Rep. 2026 May 4;9(5):e72481. doi: 10.1002/hsr2.72481 (PMC13139648; doi:10.1002/hsr2.72481)
Supplement: Supplementary file 1 — Figure S1: Structure of FbiB of the Mtb complexed with F420 cofactors. (A) Ribbon representation of Mtb FbiB bound cofactor F420 (PDB ID: 4XOQ). The F420 (orange) is demonstrated with displaying style of stick. (B) Residues in FbiB are close to the cofactor F420 binding site. (C) A ball and stick display were used to represent the recently identified mutant residues (purple). The BIOVIA Discovery Studio Visualizer v.4.5 program was used to acquire these images. Table S1: The effect of point mutations on the proteins (Ddn, Fgd1, FbiA, FbiB, and FbiC) stability and MIC range of mutants associated with DLM resistance against Mtb. [file HSR2-9-e72481-s001.docx]

**Molecular mechanisms of resistance and treatment efficacy of delamanid against Mycobacterium tuberculosis: A systematic review**

Md. Mahmudul Islam^1,2,3 †^, Md. Zahid Hasan^4, †^, Md. Touki Tahamid Tusar^5^, Md. Yeamin Hossain^6^, Md. Motaher Hossain^4^, Md. Abdulla Al Jubayed^4^, Md. Jubaer-Al-Abedin^4^, Sheikh Soikot^7^, Shanzida Akther^7^, Jahid Bhuyian^8^, Hafizur Rahman Gazi^9^, B. M. Mahmudul Hasan^10^, Md. Shofiul Azam^11^, Md. Enamul Haque^9^, Abdullah Al-Jubayer^9^, Md. Faruk Hasan^12^, F. M. Ali Haydar^13^, Md. Khalekuzzaman^14^, Muhammad Torequl Islam^15,16*^, Md. Sohel Hasan^3,17*^

*^1^Department of Genetic Engineering and Biotechnology, Daffodil International University, Dhaka 1216, Bangladesh; mahmudul.geb@diu.edu.bd*

*^2^Department of Microbiology, Shaheed Shamsuzzoha Institute of Biosciences, Affiliated with University of Rajshahi, Rajshahi 6212, Bangladesh*

*^3^Department of Biochemistry and Molecular Biology, Gopalganj Science and Technology University, Gopalganj 8105, Bangladesh*

*^4^Department of Microbiology, Rajshahi Institute of Biosciences, Affiliated with University of Rajshahi, Rajshahi 6212, Bangladesh;* [*zahidhasan5539@gmail.com*](mailto:zahidhasan5539@gmail.com); [*motaherhossain.mbio.ru@gmail.com*](mailto:motaherhossain.mbio.ru@gmail.com); [*aljubayednasim@gmail.com*](mailto:aljubayednasim@gmail.com); [*abedin9jubaer@gmail.com*](mailto:abedin9jubaer@gmail.com)

*^5^Department of Microbiology and Hygiene, Bangladesh Agricultural University, Mymensingh 2202, Bangladesh; touki.bge33@gmail.com*

*^6^Department of Fisheries, Faculty of Agriculture, University of Rajshahi, Rajshahi 6205, Bangladesh; yeamin.fish@ru.ac.bd*

*^7^Department of Pharmacy, Faculty of Science, University of Rajshahi, Rajshahi 6205, Bangladesh;* [*soikot.pharm30@gmail.com*](mailto:soikot.pharm30@gmail.com); [*shanzidaakther7@gmail.com*](mailto:shanzidaakther7@gmail.com)

*^8^Department of Agriculture, Rajshahi Institute of Biosciences, Affiliated with University of Rajshahi, Rajshahi 6212, Bangladesh;* [*jh97928@gmail.com*](mailto:jh97928@gmail.com)

*^9^Department of Biotechnology and Genetic Engineering, Gopalganj Science and Technology University, Gopalganj 8100, Bangladesh;* [*hafizur.17bge018@gstu.edu.bd*](mailto:hafizur.17bge018@gstu.edu.bd); [*enamul.haque@gstu.edu.bd*](mailto:enamul.haque@gstu.edu.bd); [*jubayer27.geb@gamil.com*](mailto:jubayer27.geb@gamil.com)

*^10^Department of Food and Nutrition, Barishal Home Economics College, Affiliated by University of Dhaka, Bangladesh;* [*jewelgono@gmail.com*](mailto:jewelgono@gmail.com)

*^11^Department of Food Engineering, Dhaka University of Engineering & Technology, Gazipur, Bangladesh;* [*shofiul@duet.ac.bd*](mailto:shofiul@duet.ac.bd)

*^12^Department of Microbiology, Faculty of Biological Sciences, University of Rajshahi 6205, Bangladesh;* [*faruk_geb@ru.ac.bd*](mailto:faruk_geb@ru.ac.bd)

*^13^Department of Botany, Faculty of Biological Sciences, University of Rajshahi 6205, Bangladesh;* [*fmalihaydar@gmail.com*](mailto:fmalihaydar@gmail.com)

*^14^Department of Genetic Engineering and Biotechnology, University of Rajshahi, Rajshahi 6205, Bangladesh;* [*kzaman63@ru.ac.bd*](mailto:kzaman63@ru.ac.bd)

*^15^Department of Pharmacy, Gopalganj Science and Technology University, Gopalganj 8100, Bangladesh;* [*dmt.islam@gstu.edu.bd*](mailto:dmt.islam@gstu.edu.bd)

*^16^Bioinformatics and Drug Innovation Laboratory, BioLuster Research Center Ltd., Gopalganj 8100, Bangladesh*

*^17^Department of Biochemistry and Molecular Biology, University of Rajshahi, Rajshahi 6205, Bangladesh;* [*sohel_bio@ru.ac.bd*](mailto:sohel_bio@ru.ac.bd)

**Correspondence*: [*sohel_bio@ru.ac.bd*](mailto:sohel_bio@ru.ac.bd) (M. S. Hasan) & [*dmt.islam@gstu.edu.bd*](mailto:dmt.islam@gstu.edu.bd) (M. T. Islam)

*Authors to whom correspondence should be addressed.

† These authors contributed equally to this work.


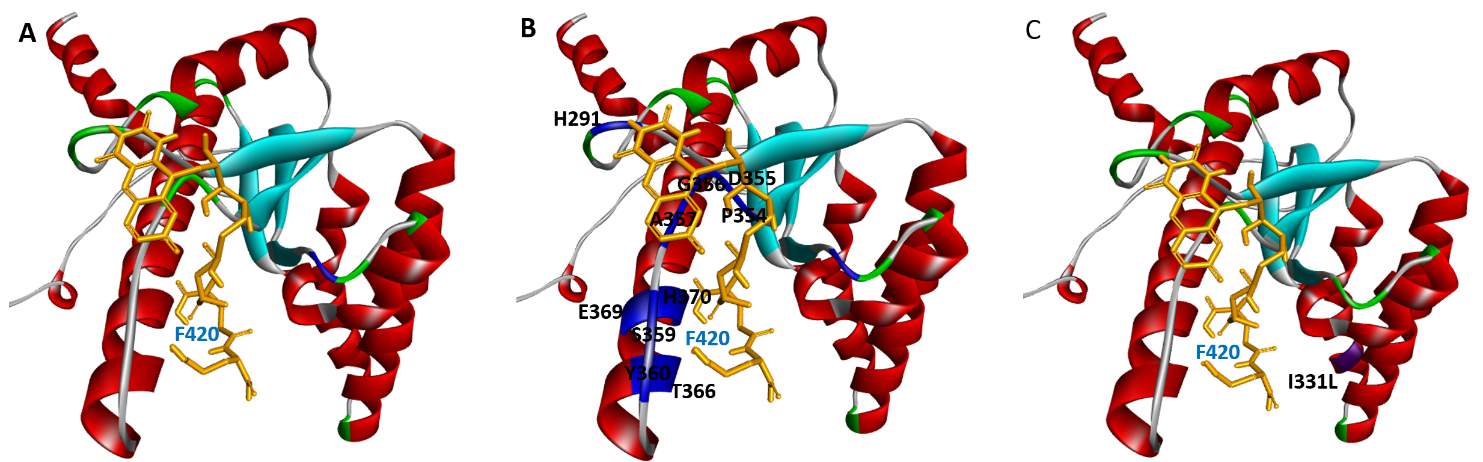


**Figure S1.** Structure of FbiB of the Mtb complexed with F420 cofactors. (**A**) Ribbon representation of Mtb FbiB bound cofactor F420 (PDB ID: 4XOQ). The F420 (orange) is demonstrated with displaying style of stick. (**B**) Residues in FbiB are close to the cofactor F420 binding site. (**C**) A ball and stick display were used to represent the recently identified mutant residues (purple). The BIOVIA Discovery Studio Visualizer v.4.5 program was used to acquire these images.

**Table S1.** The effect of point mutations on the proteins (Ddn, Fgd1, FbiA, FbiB, and FbiC) stability and MIC range of mutants associated with DLM resistance against Mtb

| **R genes** | **Point mutations** | **∆∆G (kcal/mol)*** | **S/R** | **Level of resistance** | **No. of isolates** | **Strains types** |
| --- | --- | --- | --- | --- | --- | --- |
| ddn | R23P | 0.09 | R | 0.25 μg/mL | 1 | In vitro mutants |
|  | L49P | 2.94 | R | - | 1 | Clinical |
|  | T51P | 1.63 | R | - | 2 | Clinical |
|  | G53C | 0.88 | R | 0.5 μg/mL | 1 | In vitro mutants |
|  | P63Q | 1.08 | R | >16 μg/mL | 2 | In vitro mutants |
|  | L64P | 2.21 | R | - |  | Clinical |
|  | G81S | 1.19 | R | - | 4 | Clinical |
|  | A77T | 1.57 | R | - |  | Clinical |
|  | A77P | 1.59 | R | - | 1 | Clinical |
|  | S78Y | 0.53 | R | >16 μg/mL | 1 | In vitro mutants |
|  | G81S | 1.19 | R | >1.6 mg/L | 1 | Clinical |
|  | G81D | 1.6 | R | >1.6 mg/L | 2 | Clinical |
|  | Y89S | 2.02 | R | - | 1 | Clinical |
|  | D108A | -0.34 | R | - | 1 | Clinical |
|  | T110P | 1.09 | R | - | 1 | Clinical |
|  | R112W | 0.97 | R | >16 mg/L | 1 | In vivo mutants |
|  | T115P | 0.71 | R | - | 1 | Clinical |
|  | D116E | 0.05 | R | - | 1 | Clinical |
|  | E118K | 1.39 |  | >16 μg/mL |  | In vitro mutants |
|  | T140P | 1.38 | R | - | 2 | Clinical |
|  | D141A | -0.19 | R | - | 1 | Clinical |
| fgd1 | K9N |  | R | 0.5 μg/mL | 1 | In vivo mutants |
|  | L70R |  | R | 16 μg/mL | 4 | In vitro mutants |
|  | M93R |  | R | 16 μg/mL | 1 | In vitro mutants |
|  | G191D |  | R | > 16 μg/mL | 1 | In vivo mutants |
|  | W284S |  | R | >16 μg/mL | 1 | In vitro mutants |
|  | G304V |  | R | >16 μg/mL | 1 | In vitro mutants |
|  | L321P |  | R | 0.25 μg/mL |  | In vitro mutants |
| fbiA | G8A | 1.57 | R | 0.24 mg/L | 1 | Clinical |
|  | G8S | 1.71 | R | 1 μg/mL | 1 | In vitro mutants |
|  | D49G | 0.47 | R | > 16 μg/mL | 1 | In vivo mutants |
|  | C65R | 2.3 | R | 0.25 μg/mL | 1 | In vitro mutants |
|  | Q120P | 3.57 | R | > 16 μg/mL | 1 | In vivo mutants |
|  | L143P | 1.89 | R | >16 μg/mL | 1 | In vitro mutants |
|  | A199E | 1.15 | R | 1 μg/mL | 1 | In vitro mutants |
|  | S219G | 0.35 | R | 0.03 μg/mL | 1 | In vivo mutants |
|  | E249K | 0.45 | R | > 16 | 1 | Clinical |
|  | D286A | 3.08 | R | > 16 μg/mL | 1 | In vivo mutants |
|  | L308P | 1.57 | R | > 16 μg/mL | 1 | In vivo mutants |
| fbiB | L15P | 1.43 | R | 0.125 μg/mL | 1 | In vivo mutants |
|  | L173P | 1.38 | R | 0.125 μg/mL | 1 | In vivo mutants |
|  | I331L | 0.79 | R | 16 μg/mL | 1 | In vitro mutants |
|  | G194D |  | R | 1 μg/mL | 1 | In vivo mutants |
|  | G258D |  | R | >16 μg/mL | 1 | In vitro mutants |
|  | L377R |  | R | 8-16 μg/mL | 2 | In vitro mutants |
|  | L377P |  | R | >16 μg/mL | 1 | In vivo mutants |
|  | C562W |  | R | >16 μg/mL | 1 | In vivo mutants |
|  | E598Q |  | R | 16 μg/mL | 1 | In vitro mutants |
|  | G604C |  | R | 16 μg/mL | 1 | In vitro mutants |
|  | K684T |  | R | >16 μg/mL | 1 | In vivo mutants |
|  | H698D |  | R | >16 μg/mL | 1 | In vitro mutants |
|  | A827G |  | R | >16 μg/mL | 1 | In vivo mutants |
|  | G839A |  | R | - | 1 | Clinical |

***** The free energy (∆∆G) was calculated for the point mutations in the available protein structures (Ddn, Fgd1, FbiA, FbiB, and FbiC) by using the PremPS methods
